# Automated longitudinal monitoring of *in vivo* protein aggregation in neurodegenerative disease *C. elegans* models

**DOI:** 10.1186/s13024-016-0083-6

**Published:** 2016-02-09

**Authors:** Matteo Cornaglia, Gopalan Krishnamani, Laurent Mouchiroud, Vincenzo Sorrentino, Thomas Lehnert, Johan Auwerx, Martin A. M. Gijs

**Affiliations:** Laboratory of Microsystems, EPFL, CH-1015 Lausanne, Switzerland; Laboratory for Integrative and Systems Physiology, EPFL, CH-1015 Lausanne, Switzerland

**Keywords:** *Caenorhabditis elegans*, Neurodegenerative disease, Amyotrophic lateral sclerosis (ALS), Huntington disease (HD), Doxycycline treatment, Protein aggregation, Longitudinal time-resolved analysis, High-resolution imaging, Worm immobilization, Temperature control, Microfluidics

## Abstract

**Background:**

While many biological studies can be performed on cell-based systems, the investigation of molecular pathways related to complex human dysfunctions – e.g. neurodegenerative diseases – often requires long-term studies in animal models. The nematode *Caenorhabditis elegans* represents one of the best model organisms for many of these tests and, therefore, versatile and automated systems for accurate time-resolved analyses on *C. elegans* are becoming highly desirable tools in the field.

**Results:**

We describe a new multi-functional platform for *C. elegans* analytical research, enabling automated worm isolation and culture, reversible worm immobilization and long-term high-resolution imaging, and this under active control of the main culture parameters, including temperature. We employ our platform for *in vivo* observation of biomolecules and automated analysis of protein aggregation in a *C. elegans* model for amyotrophic lateral sclerosis (ALS). Our device allows monitoring the growth rate and development of each worm, at single animal resolution, within a matrix of microfluidic chambers. We demonstrate the progression of individual protein aggregates, i.e. mutated human superoxide dismutase 1 - Yellow Fluorescent Protein (SOD1-YFP) fusion proteins in the body wall muscles, for each worm and over several days. Moreover, by combining reversible worm immobilization and on-chip high-resolution imaging, our method allows precisely localizing the expression of biomolecules within the worms’ tissues, as well as monitoring the evolution of single aggregates over consecutive days at the sub-cellular level. We also show the suitability of our system for protein aggregation monitoring in a *C. elegans* Huntington disease (HD) model, and demonstrate the system’s ability to study long-term doxycycline treatment-linked modification of protein aggregation profiles in the ALS model.

**Conclusion:**

Our microfluidic-based method allows analyzing *in vivo* the long-term dynamics of protein aggregation phenomena in *C. elegans* at unprecedented resolution. Pharmacological screenings on neurodegenerative disease *C. elegans* models may strongly benefit from this method in the near future, because of its full automation and high-throughput potential.

**Electronic supplementary material:**

The online version of this article (doi:10.1186/s13024-016-0083-6) contains supplementary material, which is available to authorized users.

## Background

The growing incidence of neurodegenerative diseases (NDs) urges for a complete understanding of the molecular processes underlying neurodegeneration, as a first step towards the final promise of a new class of therapeutics for these diseases. Cellular models have been exploited for some of these studies [1, 2], but the high complexity of the molecular mechanisms implicated in NDs increasingly demands *in vivo* models for the investigation of complex phenotypes, which are determined by the interplay among different tissues and pathways [3]. The nematode *Caenorhabditis elegans* represents a very convenient model organism for such *in vivo* tests, mainly because of its very fast life cycle, combined with the ease of its genetic manipulation and the relatively high level of conserved mechanisms between *C. elegans* and humans [4]. In the last two decades, several protein-misfolding disorders, including age-related NDs, have been successfully modeled in *C. elegans* indeed [5]; libraries of transgenic worms are currently available for the research of the molecular mechanisms underlying Alzheimer’s, Parkinson’s and Huntington’s diseases, as well as ALS [3]. Transgenic expression of disease genes in *C. elegans* is typically visualized via fluorescently tagged proteins within its transparent tissues. In most of the NDs, specific proteins self-assemble into aggregated species and cellular toxicity can be induced by the protein misfolding and aggregation process itself [3]. Therefore, the spatio-temporal-resolved observation of protein expression and aggregation, associated with the quantification and localization of these aggregates is a key analytical method for the *in vivo* monitoring of disease evolution. Unfortunately, conventional *C. elegans* handling and imaging techniques do not allow accurate monitoring of aggregate progression in individual worms over time, since nematodes are typically cultured in large populations on agar plates and irreversibly immobilized by means of anesthetics for high-resolution imaging.

The advent of microfluidics within the *C. elegans* research community is progressively revolutionizing the field [6–9]. In particular, several miniaturized devices proved their potential in neurobiology studies, such as the investigation of *C. elegans* oxygen sensation [10], olfactory [11] and chemosensory [12] neuronal activity, exploratory and learning behavior [13], neurotoxin-induced responses [14], neuromuscular function [15], and nerve regeneration [16, 17]. In the neurodegeneration research field, Càceres et al. [18] recently proposed a microscale system for high-throughput visual screens on worms. This system exploited a curved microchannel geometry to trigger the positioning of nematodes into lateral orientations and facilitate the inspection of D-type motor neurons. Although this device allowed efficiently screening mutants carrying neurodegenerative defects, it did not permit longitudinal monitoring of the worms. Other microfluidic platforms have instead demonstrated the feasibility of continuous worm culture and observation. For example, Krajniak et al. [19] showed the microfluidic culture of L1-L3 larvae over periods of 12–36 h, whilst introducing a method for worms’ reversible immobilization based on a thermo-sensitive sol–gel transition. Other studies demonstrated the applicability of this immobilization method in different microfluidic formats [20–22]. However, protein aggregation monitoring within ND disease models typically requires worm culture and repeated high-resolution imaging of the same worm over significantly longer time periods (e.g. > 3 days). This imposes severe requirements in terms of system robustness and automation, related to the simultaneous and strict control of environmental conditions, like worm feeding, fluidic exchanges, temperature of the microfluidic environment, etc.. In this perspective, Rohde et al. [23] demonstrated an elegant automated system for *in vivo* time-lapse imaging and high-throughput screening of *C. elegans* in standard multiwell plates, which employed an in-well cooling apparatus for reversible worm immobilization. However, the use of this device for protein aggregation monitoring at single animal resolution is less trivial, as it did not have microfluidics on-board and could not exploit brightfield transmission microscopy as analytical tool. Here we introduce a microfluidic-based methodology for long-term and high-resolution monitoring of protein aggregation and automated analysis of *C. elegans* ND models. Specifically, we demonstrate the feasibility of *in vivo* observation, over 4 days at single animal resolution, of SOD1 aggregation in the AM725 *C. elegans* transgenic strain, which we use as a biological model system for the investigation of the human ALS disease. This is enabled by our microfluidic platform, which co-integrates the following options and functionalities: (i) a method for fast confinement of worms of desired age in microfluidic chambers, by means of pure passive hydrodynamics with no need of any active components, such as integrated valves; (ii) a technique for continuous worm feeding and progeny removal, to preserve the on-chip worm identity over long-term studies; (iii) a method for reversible *C. elegans* immobilization using a hydrogel, enabling high-resolution imaging at arbitrarily selected moments of their whole lifespan; (iv) an integrated active temperature control system, both to set precise environmental conditions for *C. elegans* maintenance and to automatically steer the worm immobilization/release process; (v) a compact device assembly, readily adaptable to host different microfluidic designs and suitable for automated multi-dimensional imaging on any upright or inverted microscope.

## Results and discussion

### Device design

**Fig. 1:**
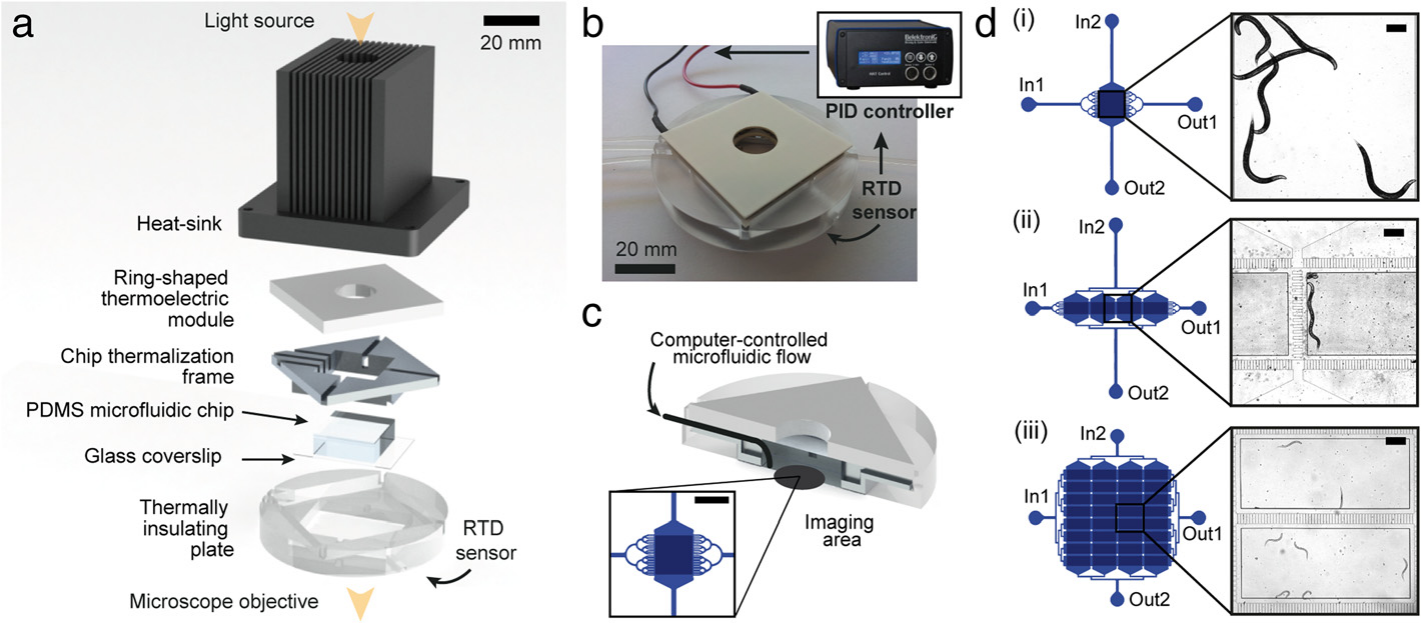
**a** Schematic representation of the main constitutive components of the microfluidic platform. **b** Photograph of the device, with schematic picture of the closed-loop temperature control system. **c** Section view of the device assembly, with a zoom on a microfluidic design within the imaging area. Scale bar = 2 mm. **d** Representative microfluidic geometries for use with the platform, featuring matrices of (i) 1, (ii) 4 and (iii) 32 worm culture chambers. Scale bars = 200 μm

Our worm culture and imaging platform features different components (Fig. 1a). Worms are manipulated inside a monolithic polydimethylsiloxane (PDMS) microfluidic chip, conceived as a simple 1-layer device and operating via pure passive hydrodynamics, with no need of any active valving system. The chip is bonded to a standard 150 μm-thick glass coverslip for accurate worm imaging through high-magnification oil immersion objectives. An aluminum frame is specifically designed to host the chip and set a well-defined temperature distribution over its whole three-dimensional (3D) geometry. Both the frame and the top of the PDMS chip are positioned in contact with a thermoelectric module used to set the temperature of the assembly. A central hole in the Peltier module allows light transmission through the PDMS chip, therefore enabling worm imaging via transmission microscopy. A well-dimensioned heat sink ensures the dissipation of excess heat produced by the thermoelectric module; a thermally insulating holder allows positioning the device on the microscope stage while preventing thermal dissipation. The entire structure is held together by screws and springs at the four corners, in order to ensure good thermal contacts throughout the stack. A resistive temperature detector (RTD), in contact with the glass substrate, is employed to sense the temperature of the microfluidic device. The signal measured by the sensor is exploited to set the power provided to the thermoelectric module in a closed-loop configuration, for active control and constant monitoring of the temperature experienced by the worms inside the chip. The feedback loop management is committed to a portable PID controller (Fig. 1b), provided with a software interface. The microfluidic flow inside the device is regulated by computer-controlled syringe pumps. The tubing connecting the chip to the external syringes are directly plugged to the sidewalls of the PDMS device. To get this peculiar configuration, we casted PDMS inside a specifically designed mold, allowing to shape its whole 3D structure (Additional file 1: Supplementary Note 1). Lateral tubing connections are employed to partially embed the tubes in the aluminum thermalization frame, therefore putting them in contact with the Peltier module as well (Fig. 1c). This allows pre-thermalizing the liquids prior to their injection inside the PDMS chip and opens the possibility of tuning the chip temperature through the injected liquid as well, as will be clarified further. Moreover, the lateral positioning of the microfluidic tubing makes our device readily suitable for imaging on both upright and inverted microscopes. Size and shape of the device holder allow perfect fit with any microscope stage or equipment compatible with standard 60 mm Petri dishes. The imaging area available for transmission microscopy has about 15 mm in diameter, corresponding to the central hole of the thermoelectric module (Fig. 1c). This represents the only geometrical constrain in the design of the PDMS chip, thus offering full versatility in using multiple microfluidic layouts. In particular, for our studies, we use three different microfluidic architectures, featuring matrices of worm culture chambers of different shapes and sizes (Fig. 1d).

### Worm arraying via passive valves

**Fig. 2:**
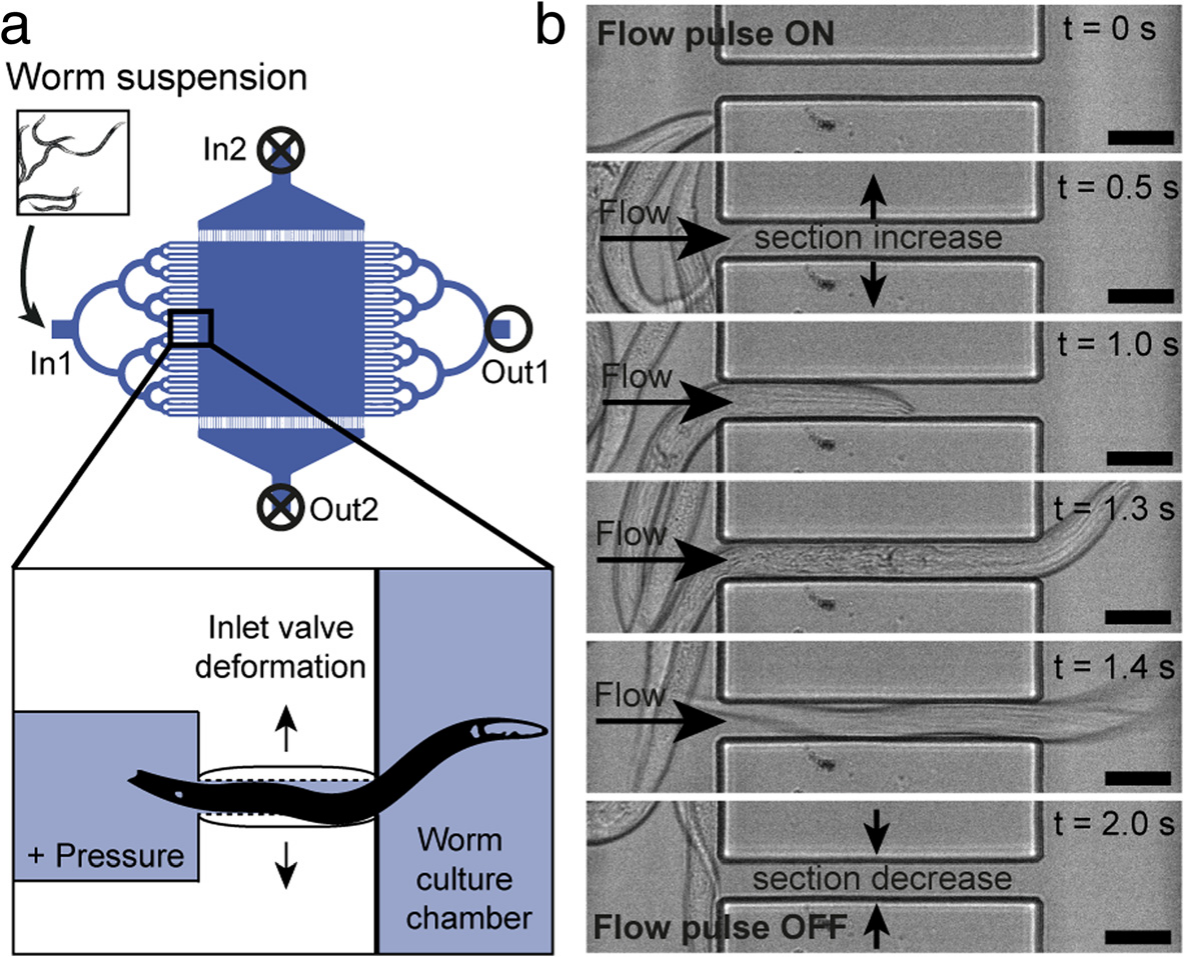
**a** Schematic representation of the worm loading process. A pressure pulse triggers the fast deformation of the PDMS valving channels and allows the injection of worms of desired size into the culture chamber. **b** Time-lapse pictures of a valving channel, as obtained from Additional file 2: Video S1, during the injection of a L1 worm in the chip of Fig. 1diii. Scale bars = 10 μm

Passive hydrodynamics allows avoiding the need of additional fluidic control layers or active valving systems, which would complicate use and design of the chip and would reduce its ease of automation. The first critical protocol steps are typically represented by synchronization, loading and distribution of the worms inside the device. In our platform, all these operations are simultaneously accomplished by simply injecting a mixed worm suspension into the microfluidic chip at a proper flow rate, along the In1-Out1 direction (Fig. 2a). We mainly worked with three different chip designs (Fig. 1d) for selecting either L1, L2/L3 or L4 larvae, as these are commonly employed stages for worm synchronization and successive analyses. Size and shape of the microfluidic channels connecting adjacent chambers along the In1-Out1 direction are designed to ensure uniform flow distribution across the chambers and allow access to the chip only to worms which are younger than the desired age – and thus smaller than a certain size. More specifically, we exploit the flexible nature of PDMS to trigger a passive valving mechanism [24] in the channels (zoom of Fig. 2a). In the “L1 design” (Fig. 1diii), only L1 worms can access the culture chambers because of their smaller size, which is compatible with the passage through microfluidic channels sizing 8 × 14 μm^2^ in section. When a 1 s pulse of 2 μL inflow is injected into the device, a slight overpressure instantaneously builds up inside the channels, causing a fast temporary increase of their section. This triggers the passage of L1 larvae through the channels (Fig. 2b and Additional file 2: Video S1). The end of the pulse results then in worm confinement inside the chambers, since each channel immediately returns to its initial shape, preventing any spontaneous passage of worms. A few subsequent inflow pulses allow distributing L1 larvae over the whole matrix of 32 chambers in a few seconds. The same principle is employed in the L4 and L2 chip designs (Fig. 1di-dii), for the automated dispensing of larvae in a single culture chamber or in the 4 chamber array. In this case microchannels (60 × 14 μm^2^ and 30 × 14 μm^2^ in section for the L4 and L2 designs, respectively) are sized as such to block the passage of adult worms, confine larvae of desired size inside the chambers by the passive valving effect, while directly washing all the smaller larvae out of the chip. Eventually, direct user control and iteration of the worm loading protocol can be used to adjust the worm distribution until a desired number of worms per chamber is obtained.

### Temperature control system design and characterization

**Fig. 3:**
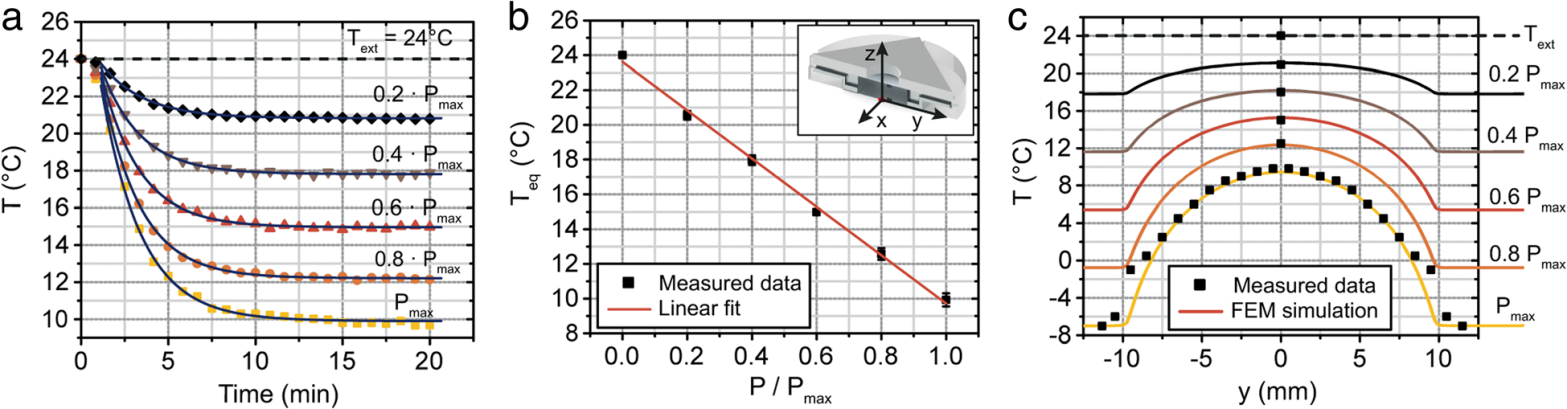
**a** Experimental characterization of the device cooling performance in “open-loop” configuration, for different cooling power applied to the thermoelectric module starting from t = 0 and an external temperature of 24 °C (temperature sensor positioned at the chip center). **b** Measured equilibrium temperature *T*_*eq*_ at the chip center (x = y = 0) for different constant cooling power and starting from an external temperature *T*_*ext*_ = 24 °C. N = 3, errors are SD. **c** Spatial temperature distribution across the chip area, both measured experimentally and simulated via FEM

We investigate the performance of our temperature control system both theoretically (Additional file 1: Supplementary Note 2) and experimentally. The setup can be operated either in “closed-loop configuration” – by means of the PID controller – or in “open-loop mode”, i.e. by providing the Peltier module with a constant electrical power. The former configuration results in fully automated control of the setup temperature, the latter option has the advantage of allowing device operation with no need of a feedback sensing system. To extract the “open loop” calibration curve of our device, we first characterize its response for different constant cooling electrical powers (Fig. 3a). In this study, the temperature is measured by means of a 1.6 × 2.5 mm^2^ Pt1000 sensor positioned on the bottom face of the glass coverslip, aligned with the center of the microfluidic chip (at x = y = 0 in the insert of Fig. 3b). Because of the reduced thickness of the glass substrate, negligible temperature variations are expected between top and bottom face of the coverslip. As expected, for different cooling power in the “open loop” configuration, the system is cooled down to specific steady-state temperatures, set by the equilibrium between thermoelectric cooling and heat convection in the surrounding air. Starting from an ambient temperature of 24 °C, stable temperatures down to 10 °C can be reached at the chip center, with a clear linear dependence on the electrical power that is applied to the Peltier module (Fig. 3b). Because of the specific geometry of the device, the spatial temperature distribution is not constant throughout the chip area, as observed via measurements at different locations from the coverslip center (Fig. 3c). By fitting these data with the corresponding simulated results, we empirically extract the heat transfer coefficient *h*, which models heat convection in our system (Equation S2). A slight deviation in the fit between measured and simulated data is observed only close to the PDMS chip edge (y = ± 10 mm) and attributed to imperfections of the contact surfaces between the chip and the metallic frame. We moreover simulate the spatial temperature distribution for different cooling powers, showing again good agreement between theoretical and experimental results (Fig. 3c). The dynamics of heat exchange in our system can be efficiently studied by normalizing the curves of Fig. 3a with respect to the external temperature *T*_*ext*_ and the steady-state temperature *T*_*eq*_ for each applied power. Normalized data prove to be independent from the electrical power and allow defining the calibration curve that describes the temperature evolution of the device in the “open loop” configuration (Additional file 1: Supplementary Note 3). Further enhancement and fine-tuning of the cooling efficiency of the device are moreover enabled by controlling the microfluidic inflow speed, which allows accurately modulating the liquid pre-thermalization (Additional file 1: Supplementary Note 4).

### Automated worm culture and imaging protocol

The cross-shape of our chips, with in- and outflows along two orthogonal directions, is designed to decouple the worms’ dispensing operation (in the In1-Out1 direction) from the worm culture and imaging protocol. Along the In2-Out2 direction, adjacent chambers are connected by narrow filters – 5 × 14 μm^2^ in section – allowing perfusion of liquids across the whole chamber matrix, while preventing any inter-chamber exchange of worms, even under over-pressure conditions. Also, after worm dispensing, the In1-Out1 flow direction can be employed during the culture experiments for evacuating the progeny of the adult worms under analysis. Each switching between the two flow directions is simply controlled by two external valves at the two chip outlets.

**Fig. 4:**
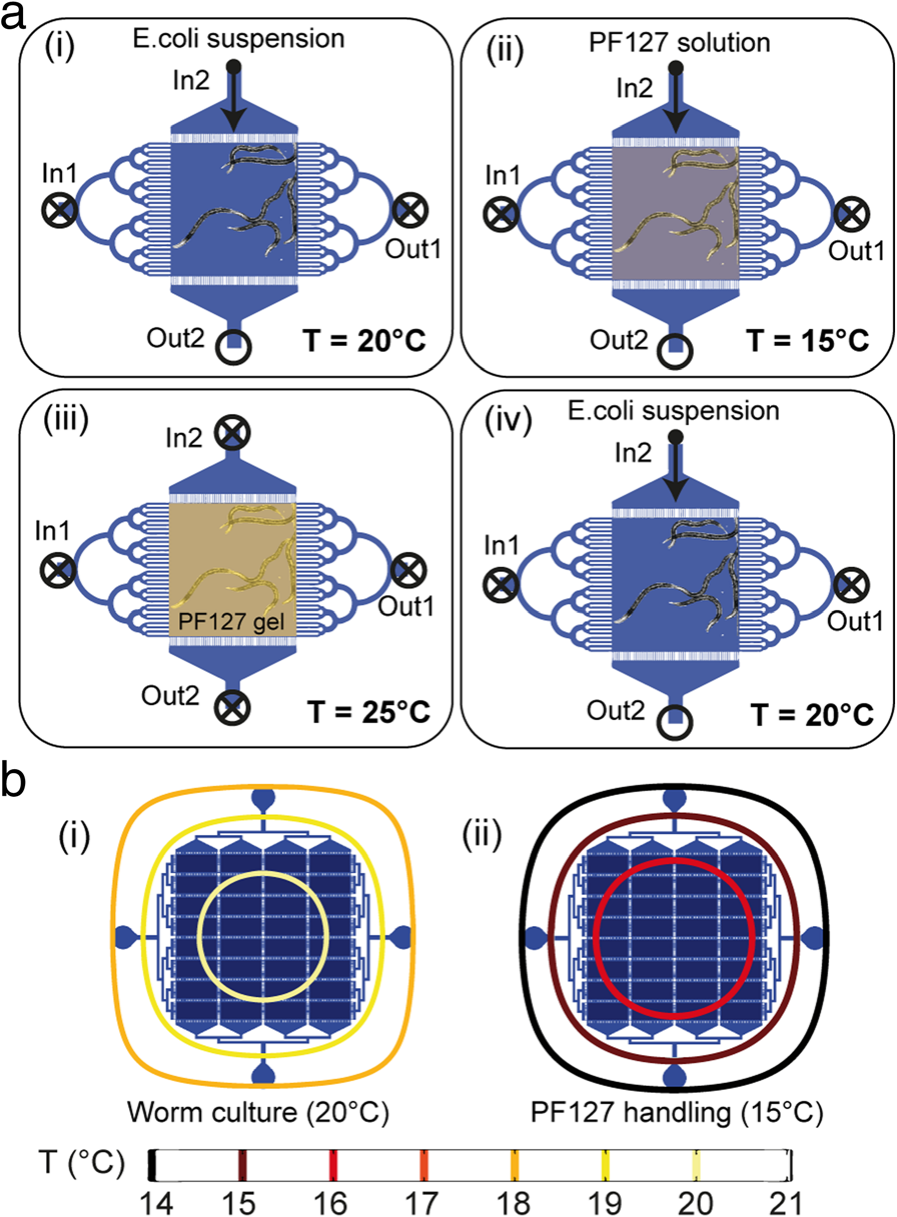
**a** Schematic representation of the iterative worm culture and imaging protocol. **b** FEM simulations of the spatial temperature distribution over the chip area (temperature contour plots) for temperature setpoints (i) at 20 °C for culture and (ii) at 15 °C for imaging

**Fig. 5:**
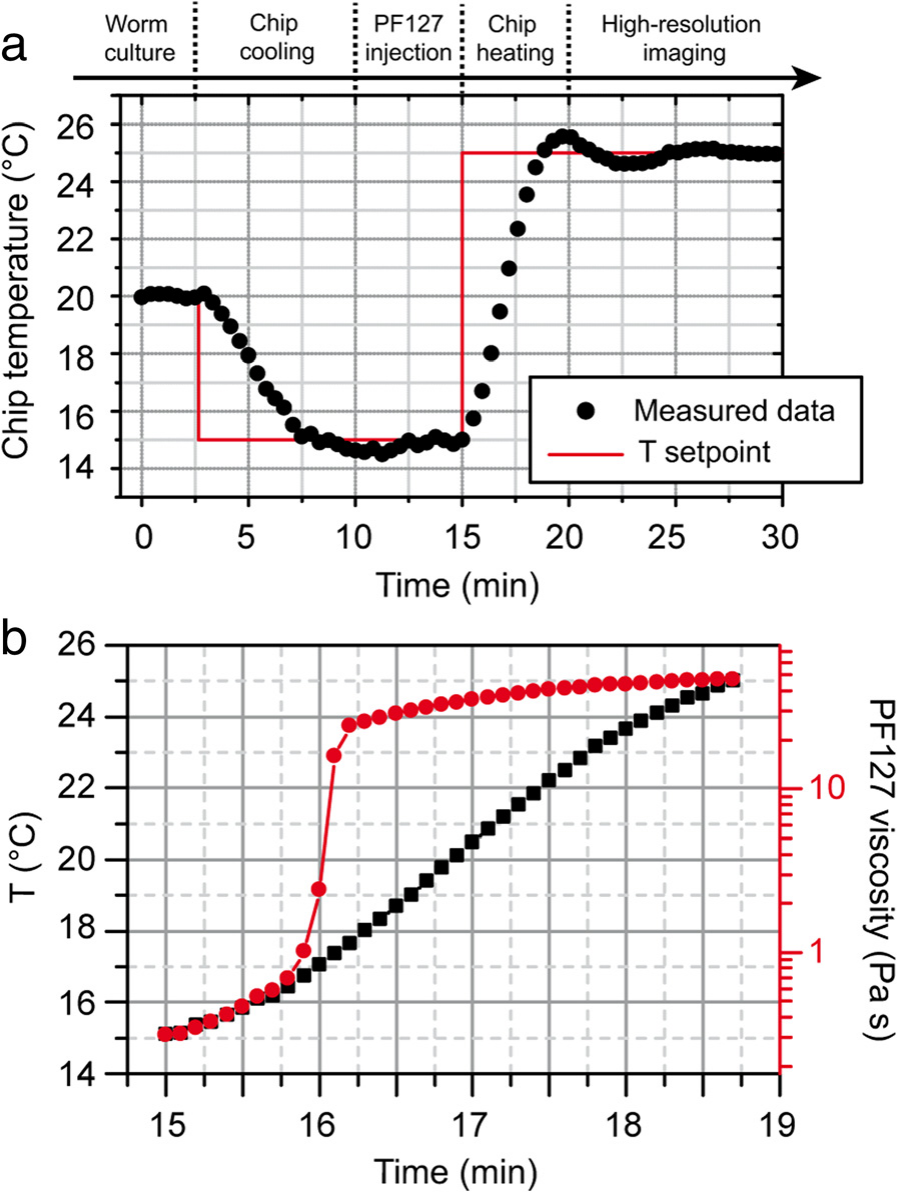
**a** Experimental temperature at the chip center during a worm culture-to-imaging transition, as managed by the active temperature control system, indicating the periods of PF127 injection and chip temperature changes. **b** Temperature rise in the 15–19 min period in more detail. The axis on the right shows the variation of PF127 solution viscosity (25 % w/v in water) during the transition from 15 to 25 °C in the device. The PF127 sol–gel transition occurs abruptly at about 17 °C, in a time window of ~1 min. Values of PF127 viscosity at the different temperatures are measured through a cone-plate viscometer

We develop a fully automated protocol for worm culture and high-resolution imaging inside the device (Fig. 4a). Worms are cultured on-chip at a desired temperature (typically in the 20–25 °C range) by operating the temperature controller in a closed-loop configuration. *Escherichia coli* suspension is perfused at desired rate along the In2-Out2 direction for worm feeding (Fig. 4ai). For screening purposes and high-throughput-like experiments, our platform can be readily used in imaging experiments with standard low-magnification microscope objectives. However, for ultimate imaging accuracy and repeatable observation of the worms through high-magnification objectives, *C. elegans* need to be temporarily immobilized in a reversible manner. To fulfil this requirement, we employ the thermoreversible gelation of a PF127 solution around the worms, as a minimally invasive *C. elegans* immobilization technique. Previously reported results, in fact, proved that both thermocycling and the exposure to PF127 for repeated imaging cycles do not alter viability, development and physiology of *C. elegans* [19]*.* Prior to imaging, the chip temperature is set at 15 °C and a liquid solution of 25 % w/v PF127 is injected into the culture chambers (Fig. 4aii). The chip temperature is then raised to 25 °C, to trigger the gelation of the Pluronic solution. This significantly increases the viscosity ensuring stable worm immobilization (Fig. 4aiii and Additional file 3: Video S2). Upon imaging, the chip temperature is brought back to 15 °C, to release the worms and wash the PF127 solution out of the chambers, by replacing it with *E. coli* suspension to restart worm culture and feeding (Fig. 4aiv). The whole protocol can be iterated many times per experiment and strongly relies on accurate temperature control, especially to trigger the worm immobilization/release process. The closed-loop temperature management system plays therefore a crucial role for the device automation. The spatial distribution of temperature over the whole chip geometry is carefully considered, to minimize inter-chamber variations and expose all the worms to the same experimental conditions. We demonstrate via FEM simulations that our device ensures temperature uniformity within 1 °C difference, even when using the largest chamber matrix, and this both during worm culture (at 20 °C) and PF127 injection and washing (at 15 °C) (Fig. 4b). PID parameters are chosen to minimize any overshoots outside of the standard temperature range for worm culture (15–25 °C) (Fig. 5a). Active temperature control allows moreover fast and accurate steering of the sol–gel transition of the PF127 solution. In our device, such a transition occurs about 1 min after the temperature setpoint shift from 15 °C to 25 °C , i.e. worms are ready for high-resolution imaging in about 2–3 min, when the viscosity of the PF127 gel reaches its highest value and guarantees worm immobilization (Fig. 5b). We experimentally determine the viscosity at different temperatures by dispensing a ~2 mL PF127 solution over the bottom plate of a cone-plate viscometer (Bohlin Gemini Malvern, UK) and measuring using a shear rate of 10 s^−1^. This value is chosen to be comparable with typical shear rates associated to the *C. elegans* swimming motion (1–20 s^−1^) [25]. Finally, we attribute a crucial role to the liquid inflow pre-thermalization as far as the PF127 injection process is concerned. Even in liquid phase, the PF127 solution behaves in fact as a very viscous non-newtonian fluid, and, as such, it is challenging to manipulate through syringes, tubes and microchannels. Pre-thermalizing the PF127 inflow in the metallic frame to temperatures even lower than 15 °C allows further reducing the solution viscosity at the chip inlet and improving the control over its injection.

### Long-term protein aggregation analysis in an amyotrophic lateral sclerosis (ALS) *C. elegans* model

**Fig. 6:**
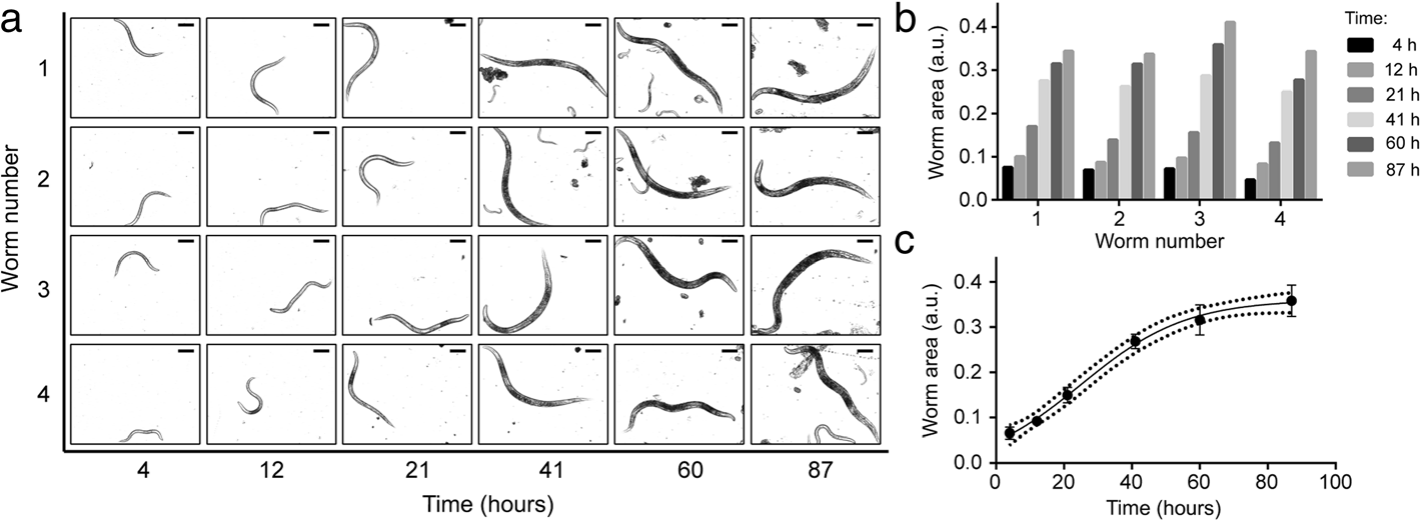
**a** Time-lapse brightfield pictures of four AM725 transgenic worms, isolated at t = 0 at the L2 larval stage in the 4 different culture chambers. Scale bars = 100 μm. **b** On-chip growth rate of the four worms over 87 h, as estimated by measuring the worm area from time-lapse pictures. **c**) Average on-chip worm growth, featuring a sigmoidal time-dependence

**Fig. 7:**
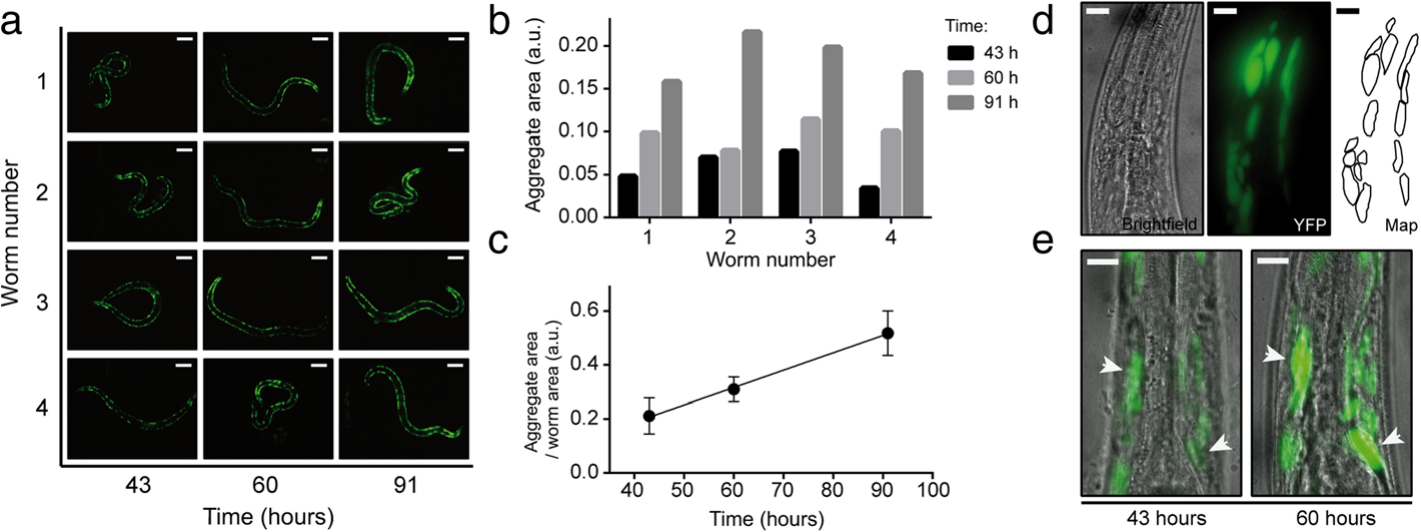
**a** Time-lapse fluorescent pictures of four AM725 transgenic worms, immobilized in a PF127 gel matrix within the culture chambers. Scale bars = 100 μm. **b** Growth rate of SOD1-YFP aggregates in the body wall muscle cells of each worm, as estimated by measuring YFP expression area across each worm’s body during their immobilization in the gel matrix. **c** Average protein aggregate/worm area over time. For each worm and each time-point, the aggregate area is normalized by the worm area to take into account the size variability of each worm. A clear time-dependent increase of these values is observed over the period from 43 to 91 h upon loading on chip (day 1 to day 3 of worm adulthood). **d** Brightfield and fluorescent images of an immobilized worm (worm 1), as taken through a 63× NA 1.4 oil immersion objective 91 h upon worm loading into the device. These pictures allow mapping the aggregate morphology at high spatio-temporal resolution. **e** Superimposed brightfield and fluorescent images of an immobilized worm (worm 4), as taken through a 63× NA 1.4 oil immersion objective 43 and 60 h upon worm loading into the device. Arrows point at specific SOD1-YFP aggregates, which can be re-identified in subsequent images and tracked over time. Scale bars = 20 μm

Upon full validation of worm viability and culture in our device (Additional file 1: Supplementary Note 5), we employ it to monitor the dynamics of protein aggregation in an ALS disease *C. elegans* model. ALS is a neurodegenerative human disease causing selective death of motor neurons. In Europe and the United States, this disease affects about 2 people per 100,000 per year [26], with average survival from onset to death of only 3–5 years and no cure currently available [27]. In ALS, such as in many other neurodegenerative diseases, cellular toxicity could be mediated by the misfolding and aggregation of specific mutant proteins [28–30]. Time-resolved imaging and quantification of these aggregates is hence a key phenotyping method to monitor disease progression. In our study, we employ an ALS *C. elegans* model expressing mutated human SOD1-YFP fusion proteins in the body wall muscle cells (AM725 transgenic worms) [31] While this model presents some limitations for investigating neurodegeneration *per se*, compelling evidence in literature already proved that *C. elegans* models expressing protein aggregation in muscle cells are useful for screening neurodegenerative phenotypes about Parkinson’s and Huntington’s diseases [32–34]. In our device, worms are loaded at the L2 stage into a 4-chamber microfluidic chip (Fig. 1dii), where their distribution is adjusted to isolate a single worm in each chamber. Automated on-chip culture of these worms is then conducted according to the previously described protocols. Correct worm feeding, development and reproduction are constantly monitored via brightfield time-lapse imaging (Fig. 6a). Automated image processing algorithms on these time-lapse pictures allow moreover extracting detailed information about the worms’ growth rate, not only at a population level, but also at single-worm resolution (Fig. 6b-c). Specifically, the area occupied by each worm is here used as a quantitative indicator of growth. Data about the growth rate of individual worms allows preserving detailed information on the development of each nematode (Fig. 6b), while the average growth trend is found to follow a well-defined sigmoidal dependence on time (Fig. 6c). At desired moments during worms’ development (e.g., in our study, 43 h, 60 h and 91 h upon worm injection), the reversible worm immobilization protocol for high-resolution imaging (see Fig. 4a) is employed. At each observation and in a matter of a few minutes, all the worms are perfectly immobilized in a PF127 gel matrix and SOD1-YFP expression can be monitored within their tissues via fluorescent microscopy through high-magnification objectives. A first analysis at 10× magnification allows observing protein aggregates within the whole body of each worm (Fig. 7a). Aggregation patterns observed in our system are in line with previously reported results: while wild-type SOD1 is known to exhibit only diffuse fluorescence in body wall muscle cells, mutant SOD1 proteins present punctuated fluorescent patterns [31]. Interestingly, we find that the temporal evolution of SOD1-YFP aggregation features some observable worm-to-worm variability (Fig. 7b), and follows an increasing trend in the considered temporal window (43–91 h after on-chip loading at the L2 stage) (Fig. 7c).

A second set of studies is then conducted by imaging the immobilized worms through a 63× oil immersion objective (NA 1.4). Many aggregates in AM725 worms, unlike in other analogous SOD1-transgenic strains – e.g. p*Unc-54*::SOD1-G85R::YFP (G85R) and p*Unc-54*::SOD1- G93A::YFP (G93A) –, appear as irregular, elongated foci [31]. This feature could be observed before by confocal imaging of paralyzed worms. We are now able to confirm this observation in alive immobilized worms and provide a precise sub-cellular mapping of their protein aggregation pattern at high spatio-temporal resolution by using a standard fluorescent microscope (Fig. 7d). Moreover, the possibility to take quasi-instantaneous brightfield and fluorescent pictures in our chip allows accurately locating each fluorescent signal inside the *C. elegans* body. In combination with reversible worm immobilization, this opens the possibility of following the temporal evolution of protein aggregation at precise locations within the worm tissues and monitoring aggregate progression *in vivo*, not only at single-worm, but even at the single-cell level (Fig. 7e).

### Long-term protein aggregation analysis in a Huntington disease (HD) *C. elegans* model

To ascertain that this approach is not specific only for the ALS model, we monitor the dynamics of protein aggregation in a different *C. elegans* model of neurodegenerative diseases, i.e. a Huntington disease (HD) model (Additional file 1: Supplementary Note 6). More particularly, we employ a HD model expressing YFP fused to stretches of 35 glutamine residues (AM140 transgenic strain) [35]. Our system allows the observation of distinct aggregation patterns for this model and allows following the long-term evolution of both aggregate size and number, with results in line with what has been previously reported [35]. Furthermore, the specificity of the approach to monitor the aggregates is validated by using a strain expressing the YFP only in the body wall muscles (AM134 strain) and showing only a diffuse fluorescence in these tissues [35].

### Modifying long-term protein aggregation in an ALS *C. elegans* model by doxycycline treatment

**Fig. 8:**
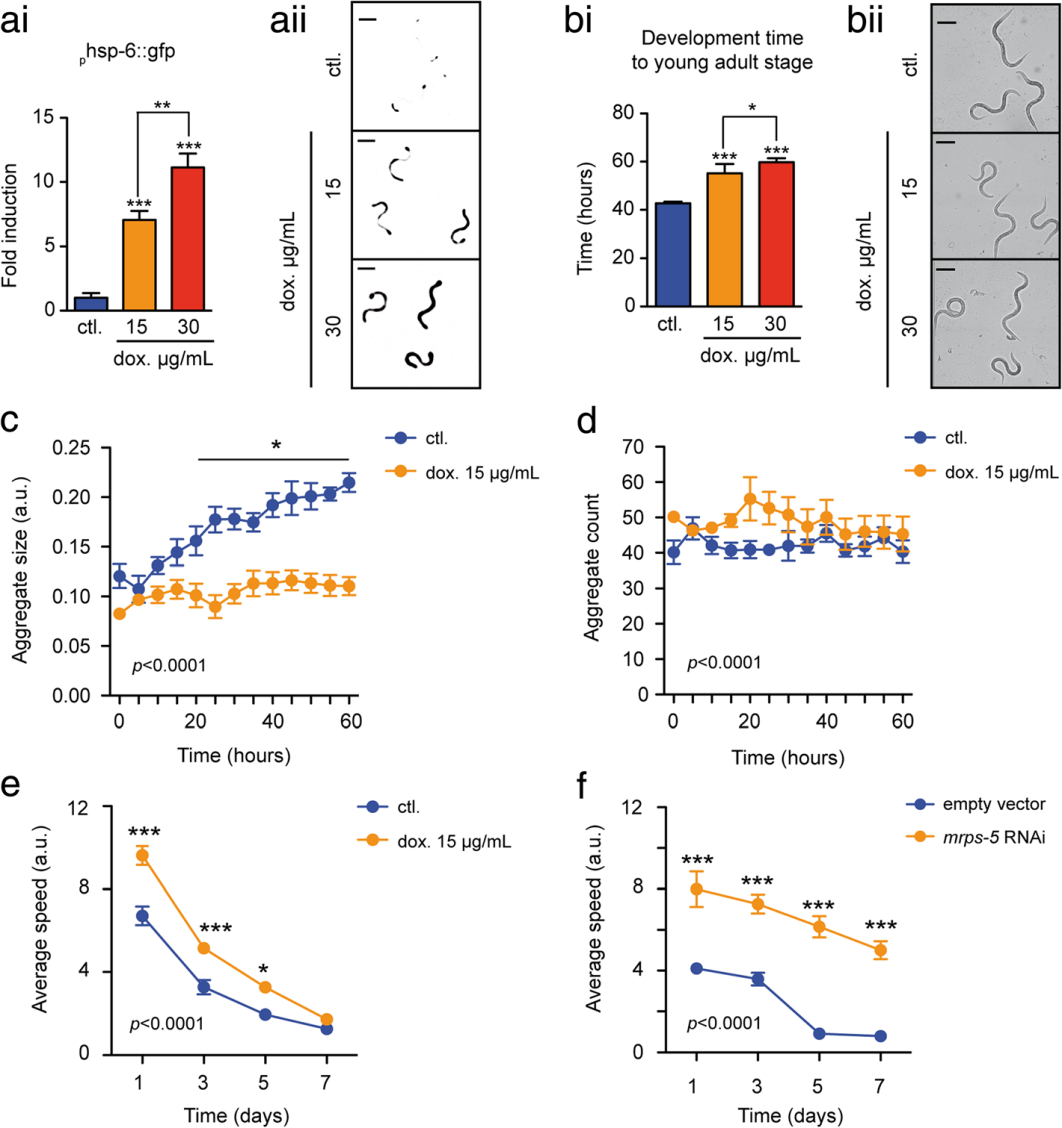
**a**-**b** Analysis of the efficacy of doxycycline treatment in our microfluidic system. (ai) Quantification of *hsp-6::gfp* expression in young adult SJ4100 worms, treated on-chip with doxycycline at concentrations of 0, 15 and 30 μg/mL, starting from the L1 stage. (aii) Representative fluorescent pictures of young adult worms upon image thresholding, as used for the quantification of *hsp-6::gfp* expression. Scale bars = 200 μm. (bi) Quantification of the development time to the young adult stage in SJ4100 worms, treated on-chip with doxycycline at concentrations of 0, 15 and 30 μg/mL, starting from the L1 stage. (bii) Representative brightfield pictures of young adult worms, as used for the monitoring of the development time. Scale bars = 200 μm. **c**-**d** Temporal evolution of (**c**) the average size and (**d**) the average number of aggregates counted in AM725 worms, over a 60 h period upon the onset of young adult stage (set as t = 0). Different aggregation profiles are observed between untreated AM725 worms and the AM725 worms treated on-chip with doxycycline at 15 μg/mL concentration, starting from the L1 stage. Graphs are expressed as mean + SEM (N = 5). **e**-**f** Temporal evolution of worms’ motility over 7 days of adulthood, as observed on NGM plates for (**e**) doxycycline-treated vs. untreated AM725 worms and (**f**) *mrps-5* RNAi-treated vs untreated AM725 worms

ALS is the most common motor neuron disease in adults, causing the selective loss of the spinal and cranial motor neurons cells that directly connect the brain to muscles. The disease is characterized by rapidly progressive paralysis and death from respiratory failure, typically within 2–3 years of symptom onset [36]. There are currently no effective cures for ALS, although Riluzole was found to slow the rate of progression and prolongs survival by 3 months [36]. Mitochondrial accumulation of misfolded mutant SOD1 has been proposed as one possible trigger of motor neuron death [37]. Mitochondrial degeneration [38], vacuolization and swelling [39] are pathological features of both familial human ALS cases and mutant SOD1 mouse models. Hence, damaged or dysfunctional mitochondria are common in most familial ALS cases and could represent a potential therapeutic target for treatment of this disease. Activation of the mitochondrial unfolded protein response (UPR^mt^) recently emerged as an interesting approach to restore a pool of healthy, functional mitochondria in stressed animals and maintain organism health [40]. Indeed, genetic or pharmacological inductions of UPR^mt^ can extend the lifespan in various model organisms, including worms [41–43]. Beneficial effects of UPR^mt^ rely on the massive expression of mitochondrial chaperones that help to repair the damages caused in the organelle [44]. Doxycycline –an antibiotic belonging to the tetracycline family– proved to be beneficial for certain physiological aspects, like for increasing the motility in worms and flies, and extending the lifespan of worms through the activation of UPR^mt^ [42, 45]. To determine whether UPR^mt^ could prevent the ALS progression in *C. elegans*, we treat worms with doxycycline and monitor the aggregate formation. First, in order to evaluate the efficacy of doxycycline treatment in our microfluidic system, we need to monitor the induction of the UPR^mt^. We therefore use a transgenic strain of worms that reports on the activity of the UPR^mt^ with integrated GFP genes driven by the regulatory DNA region of the mitochondrial chaperone *hsp-6* [46]. In these transgenic worms, an increase of *hsp-6::gfp* expression is indicative of the presence of a mitochondrial stress and the subsequent induction of the UPR^mt^ [46]. As previously observed on solid nematode growth media (NGM) plates [42], doxycycline dose-dependently also induces the expression of the *hsp-6::gfp* on-chip, revealing the activation of the UPR^mt^ (Fig. 8a). At the same time, doxycycline also induces a significant growth delay, which is another known physiological effect produced by the antibiotic (Fig. 8b) [45]. Based on these first observations obtained from the *hsp-6::gfp* expressing worms, we decide to use a concentration of 15 μg/mL doxycycline in our microfluidic experiments on the ALS worm model, as (1) this concentration is comparable to the concentrations showing a significant effect on the worm lifespan and on the UPR^mt^ on solid plates, and (2) the impact on the growth of this concentration is minor. Moreover, we treat ALS worms with doxycycline and compare the aggregation pattern with that of an untreated population. Strikingly, doxycycline treatment suppresses almost completely the size expansion of the aggregates, without affecting their number (Fig. 8c-d).

Protein aggregation diseases are often associated with movement disorders, and worm models of neurodegenerative diseases also recapitulate this feature [31, 35, 47–49]. Interestingly, doxycycline treatment significantly slows down the progressive loss of motility in the ALS strain, as observed on NGM plates (Fig. 8e). Since doxycycline is known to affect the mitochondrial translation in eukaryote systems [45], we then employ a genetic approach to impact on the mitochondrial translation machinery. We silence by RNAi the gene *mrps-5* (mitochondrial ribosomal protein S5), an important molecular actor for the mitochondrial translation [42], and we could observe similar effects on the motility in the ALS model (Fig. 8f). All together, these results show that doxycycline can prevent the size expansion of aggregates in a *C. elegans* model of ALS, and this improvement correlates with a delay in the loss of motility.

## Conclusions

We introduce a new analytical technique and device for automated time-resolved studies on *C. elegans* nematodes down to single-worm resolution. Our platform is based on a multi-functional approach, where several functionalities are integrated into a single miniaturized device, to allow fully automated worm analyses. The device is moreover designed to be compatible with different microfluidic designs and readily suitable for different sets of studies.

In our platform, *C. elegans* nematodes are loaded into a microfluidic chip, where they are directly distributed among a set of culture chambers via a “passive valving” method. Geometrical constraints on the chip allow retaining only worms of desired size inside the device, whereas an automated on-chip culture protocol is established to ensure their correct feeding and development. Active control of the chip temperature ensures moreover running worm cultures at desired temperatures, with minimal variation throughout long-term analyses. For screening purposes and high-throughput-like experiments, our platform can be readily used in imaging experiments with standard low-magnification microscope objectives. Moreover, to allow longitudinal high-resolution imaging of the worms, we optimize an automated procedure for reversible worm immobilization on-chip. This protocol is based on the thermoreversible gelation of PF127 polymer inside the device, managed by the closed-loop temperature control system as well. Any worms’ progeny is periodically washed out of the chip, with no risk of mixing the identities of the worms under analysis. Therefore, tests at single-worm resolution can be easily performed on our platform. Finally, all the microfluidic designs used in our studies are conceived in a “chamber-matrix” format, which allows easy automation of the imaging process as well.

We fully characterize the different functionalities of our platform, both theoretically and experimentally. We demonstrate fast and precise temperature management on the device and provide calibration curves for its use both in open-loop mode and in closed-loop configuration. We characterize the different integrated worm handling protocols – i.e. on-chip worm loading, feeding, immobilization, imaging – and provide details for their use on the platform. We then employ our device to tackle the challenging task of analyzing the dynamics of protein aggregation in ALS worm models over long-term experiments. Our results show that the device ensures reliable culture and reproducible growth rate of the worms over several days. The possibility of isolating single worms in separated chambers allows collecting population statistics, while preserving at the same time all the information related to the single nematodes under test. For high-resolution imaging experiments, we employ the on-chip immobilization protocol to temporarily immobilize the worms in a reversible way and periodically collect data about protein aggregation in their tissues via high-resolution fluorescent imaging. Our results show that the amount of SOD1-YFP aggregates in an ALS *C. elegans* model (AM725 transgenic worms) linearly increases over the whole analyzed period (i.e. day 1 to day 4 of adult life). Combined brightfield and fluorescent imaging at high magnification allows moreover mapping the geometry of the aggregates, precisely locate them within the tissues of each worm and following their progression over consecutive days. We also demonstrate the suitability of our system for protein aggregation monitoring in a *C. elegans* Huntington disease (HD) model, and demonstrate the systems’s ability to study long-term doxycycline treatment-linked modification of protein aggregation profiles in the ALS model. In fact, the relatively short period needed for the quantification of a significant increase in SOD1-YFP aggregates opens the possibility for future studies of rapid identification of ALS modifiers [50]. Because of its good performance in terms of automation and versatility, we envision that our system could be employed to address many other challenging biological questions on *C. elegans*, related in particular to the study of neurodegenerative diseases – such as Parkinson’s, and Alzheimer’s disease – which are all modelled in worms [3].

Our platform could moreover be used for studies of *C. elegans* movement disorders, for quantifying other phenotypes such as pharyngeal pumping rates, motility, etc. or more generally could be used in chemical or biological laboratories that do *in vivo* studies and analyses of multicellular organisms.

## Methods

### Chemicals and materials

Four-inch 550 μm thick Si and float glass wafers, de-ionized water (DIW) were obtained from the Center of Micro- and Nanotechnology of EPFL. GM 1070 SU-8 negative photoresist was purchased from Gersteltec (Pully, Switzerland). PDMS Sylgard 184 was acquired from Dow Corning (Wiesbaden, Germany). 1 mL borosilicate H-TLL-PE syringes were purchased from Innovative Laborsysteme GmbH (Stutzerbach, Germany). Microline ethyl vinyl acetate tube with 0.51 mm inner and 1.52 mm outer diameters was bought from Fisher Scientific (Wohlen, Switzerland). Pluronic F-127 was purchased from Sigma-Aldrich (Buchs, Switzerland). M9 buffer was obtained by adding 3 g KH_2_PO_4_, 6 g Na_2_HPO_4_, 5 g NaCl, 1 mL 1 M MgSO_4_, H_2_O to 1 l and sterilization by autoclaving. S-medium buffer was obtained by adding 10 mL 1 M potassium citrate pH 6, 10 mL trace metals solution (1.86 g disodium EDTA, 0.69 g FeSO_4_ · 7H_2_O, 0.2 g MnCl_2_ · 4H_2_O, 0.29 g ZnSO_4_ · 7H_2_O and 0.025 g CuSO_4_ · 5H_2_O, H_2_O to 1 l) 3 mL 1 M CaCl_2_, 3 mL 1 M MgSO_4_, 1 mL [50 mg/mL] carbenicillin, 0.5 mL tween 20 to 1 l S Basal (5.85 g NaCl, 1 g K_2_HPO_4_, 6 g KH_2_PO_4_, 1 mL [5 mg/mL] cholesterol, H_2_O to 1 l) and sterilization by autoclaving. Pluronic F127 solution was prepared by diluting 25 % (weight/volume) Pluronic F127 in water. Aluminum and polymethylmetacrylate (PMMA) assembly parts were fabricated at the engineering workshop of EPFL. Thermoelectric modules were bought from TE Technology, Inc. (Traverse City, MI, USA), heat sinks from Advanced Thermal Solutions, Inc. (Norwood, MA, USA) and RTD sensors from Innovative Sensor Technology AG (Ebnat-Kappel, Switzerland), while a proportional-integral-derivative (PID) temperature controller was purchased from BelektroniG GmbH (Freital, Germany).

### *C. elegans* strains and culture

*C. elegans* strains were cultured at 20 °C on NGM agar plates seeded with the *Escherichia coli* strain OP50. Strains used were wild-type Bristol N2, AM725 (rmIs290[unc-54p::Hsa-sod-1(127X)::YFP]), AM134 (rmIs126[unc-54p::Q0::YFP]), AM140 (rmIs132[unc-54p::Q35::YFP]) and SJ4100 (zcIs13[hsp-6::GFP]) and were provided by the Caenorhabditis Genetics Center (University of Minnesota). Worms were suspended in S-medium solution prior to each microfluidic experiment. For microfluidic experiments, the *E. coli* strain HT115 was suspended in S-medium at a concentration of 1.4 × 10^9^ cells/mL. Bacterial feeding RNAi experiments were carried out as described [51]. The clone used was *mrps-5* (E02A10.1). For on-plate motility assays, doxycycline was added at the indicated concentration just before pouring the plates. Animals were exposed to compounds from eggs until the day of the experiment.

### Motility assays

*C. elegans* movement was recorded for 45 s at different days of adulthood using a Nikon DS-L2/DS-Fi1 camera and controller setup, attached to both a computer and a standard brightfield microscope. Five plates of worms, with 10 worms per plate were measured in each condition. Using these video recordings, the movement traces of worms during all recording periods were calculated by following the organism centroids using a modified version of the Parallel Worm Tracker for MATLAB [52]. The average worm speed during the recording periods was then calculated for each plate and each condition.

### Fabrication of the microfluidic chips

Microfluidic devices were prepared by soft lithography [53] using 2-layer SU-8 molds. Briefly, conventional photolithography was used to pattern a 14 μm-thick layer of SU-8 photoresist on 4-in. wafers. A ~110 μm-thick layer of SU-8 was then patterned on top of the first one. Layer thicknesses were confirmed by mechanical profilometer measurements. The silicon mold was then diced in 20 mm × 20 mm microchips, which were inserted at the bottom of an aluminum/PMMA mold for PDMS casting (Additional file 1: Supplementary Note 1). 1.5 mm diameter steel pins were used to define the lateral connections of the device for the external tubing insertion. A liquid PDMS mixture (10:1 base:cross-linker weight ratio) was degassed, injected into the mold and cured at 100 °C for 1 h. Upon extraction from the mold, each PDMS chip was bonded by plasma-activation to a 150 μm-thick, 32 × 24 mm^2^ glass coverslip. The chip was then connected to external tubing and enclosed in the device assembly as reported in Fig. 1a.

### Image acquisition and processing

The microfluidic platform was placed within an inverted microscope (Axio Observer, Zeiss) equipped with two illumination systems: (i) a precisExcite High-Power LED illumination system (Visitron, Puchheim, Germany) for brightfield imaging and (ii) a Lambda DG4 illumination system (Sutter instruments, Novato, CA, USA) for fluorescence imaging. The microscope had a motorized xy-stage and the automated imaging process was controlled using VisiView Premier Image acquisition software (Visitron, Puchheim, Germany). Images were acquired through a Hamamatsu Orca-ER CCD camera (Hamamatsu, Solothurn, Switzerland). Image processing was performed with Fiji software (http://imagej.nih.gov/ij; version 1.47b). In particular, worm areas were measured by processing time-lapse brightfield pictures as follows. Each frame was first converted to a binary image by applying a threshold to the full stack of time-lapse images and transforming it into a set of binary masks. Each stack of masks was then analyzed using the “particle analysis” Fiji plugin, which allows directly extracting area values for each picture in the stack. The same method was then applied to the stacks of fluorescence images, in order to calculate aggregate area values. In this case, we applied a systematic thresholding algorithm which was based on a variational approach, assuring that all aggregates in an image were effectively counted (i.e. by not setting the threshold too low), while not resulting in artificial size reduction of the aggregates (which would be the case by fixing a too high threshold). The “particle analysis” plugin allowed measuring the number and the average size of the aggregates identified in each picture.

### Supporting information

Additional material is available: supplementary notes on (i) 3D PDMS chip casting with lateral fluidic connections; (ii) temperature control system: theoretical considerations; (iii) heat exchange dynamics in the device; (iv) inflow pre-thermalization study; (v) worm viability and culture tests; (vi) protein aggregation analysis in a Huntington disease *C. elegans* model. Supplementary videos on: (i) L1 worm loading via passive valves, and (ii) AM725 worm immobilization by PF127 gelation.

## Additional files

Additional file 1:
**Supplementary Information.** (PDF 1126 kb) Additional file 2: Video S1. L1 worm loading via passive valves. (AVI 62870 kb) Additional file 3: Video S2. AM725 worm immobilization by PF127 gelation. (AVI 8800 kb)
