# Supplementary material for: Automated longitudinal monitoring of in vivo protein aggregation in neurodegenerative disease C. elegans models
Source: Mol Neurodegener. 2016 Feb 9;11:17. doi: 10.1186/s13024-016-0083-6 (PMC4746889; doi:10.1186/s13024-016-0083-6)
Supplement: Additional file 1: — Supplementary Information. (PDF 1126 kb) [file 13024_2016_83_MOESM1_ESM.pdf]

Supplementary Information

Automated longitudinal monitoring of *in vivo* protein aggregation in neurodegenerative disease *C. elegans* models

Matteo Cornaglia[a], Gopalan Krishnamani[a], Laurent Mouchiroud[b], Vincenzo Sorrentino[b], Thomas Lehnert[a], Johan Auwerx[b], and Martin A. M. Gijs[a]*

[a] Laboratory of Microsystems, EPFL, CH-1015 Lausanne, Switzerland.

[b] Laboratory for Integrative and Systems Physiology, EPFL, CH-1015 Lausanne, Switzerland.

* Author to whom correspondence should be addressed. E-mail: matteo.cornaglia@epfl.ch

Supplementary Notes

**Supplementary Note 1**	3D PDMS chip casting with lateral fluidic connections.
**Supplementary Note 2**	Temperature control system: theoretical considerations.
**Supplementary Note 3**	Heat exchange dynamics in the device.
**Supplementary Note 4**	Inflow pre-thermalization study.
**Supplementary Note 5**	Worm viability and culture tests.
**Supplementary Note 6**	Protein aggregation analysis in a Huntington disease *C. elegans* model.

Supplementary Videos, as available on the journal's website.

**Supplementary Video 1**	L1 worm loading via passive valves.
**Supplementary Video 2**	AM725 worm immobilization by PF127 gelation.

Supplementary Note 1. 3D PDMS chip casting with lateral fluidic connections.

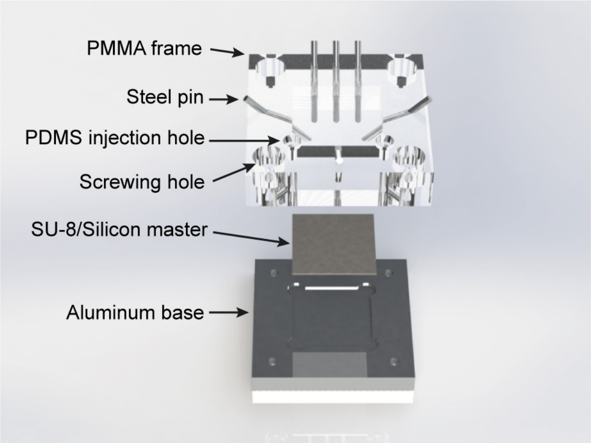

Figure SI1 Schematic representation of the casting mold designed to shape the whole 3D geometry of our PDMS microfluidic chips. The SU-8/silicon master corresponding to the desired microfluidic design is positioned inside an aluminum base, which is then sealed to a polymethylmethacrylate (PMMA) frame using 4 M3 screws. 1.5 mm diameter steel pins are inserted into the PMMA frame through dedicated holes, machined at 45° angle with respect to the master mold. The flat base of each pin contacts the SU-8 structures at the position of each inlet (or outlet). This specific configuration is conceived to achieve lateral microfluidic connections entering the sidewalls of the chip, leaving hence free access for observation and manipulation both from the top and the bottom faces of the chip. PDMS is injected inside the casting mold through the “PDMS injection hole” using a syringe. The whole assembly is then positioned between two parallel hot plates at 100 °C for PDMS curing, using the aluminum base part to ensure good thermal exchange with the hot plates, so that the liquid PDMS correctly polymerizes.

Supplementary Note 2. Temperature control system: theoretical considerations.

We investigate the performance of our temperature control system both theoretically and experimentally. The setup can be operated either in “closed-loop configuration” – by means of the PID controller – or in “open-loop mode”, i.e. by providing the Peltier module with a constant electrical power. The former configuration results in fully automated control of the setup temperature, the latter option has the advantage of allowing device operation with no need of a feedback sensing system.

When electrical power is supplied to the thermoelectric module, heat exchange is induced through the assembly, resulting in the heating or cooling of the chip, for positive and negative

electrical powers, respectively. The temperature distribution achieved in the device satisfies the following equation in time:

$$\rho C_p \frac{\partial T}{\partial t} + \nabla(-k\nabla T) = Q \qquad \text{(S1)}$$

with density ρ, heat capacity C_p, and thermal conductivity k, varying according to the material (a scalar or a tensor for an isotropic or anisotropic material, respectively). In our case the temperature T is modulated by the heat source Q (unit: W/m^3), represented by the Peltier module. Further heat exchange occurs then via convective heat transfer at the boundaries of the assembly through the surrounding air, according to:

$$q = h(T_{ext} - T) \qquad \text{(S2)}$$

where q is the convective heat flux through a boundary (unit: W/m^2), h is the heat transfer coefficient and T_{ext} the temperature of the ambient air, far from the boundary. Because of the composite geometry of our device, including different materials with complex 3D shapes, equations (S1) and (S2) are best solved via 3D Finite Element Method (FEM) modeling, as detailed in the main text.

Supplementary Note 3. Heat exchange dynamics in the device.

The dynamics of heat exchange in our system can be efficiently studied by normalizing the curves of Figure 3a with respect to the external temperature T_{ext} and the steady-state temperature T_{eq} for each applied power. Normalized data prove to be independent from the electrical power and allow defining the calibration curve that describes the temperature evolution of the device in the "open loop" configuration (Figure SI2). The same result can be obtained via FEM by plotting the temperature at the chip center using a time-dependent simulation. In this analysis, the coefficient of performance (COP) of the thermoelectric module needs to be carefully taken into account, with COP typically defined as the ratio between the effective cooling power and the supplied electrical power. In our case, when the maximum allowed electrical power is provided to the thermoelectric module (P_{max} ~ 40 W), its COP is inversely proportional to the temperature difference created between its two plates (information extracted from the datasheet of the thermoelectric module). By including this specific dependence in our simulation, we obtain a theoretical calibration curve in very good agreement with the experimental data (Figure SI2). The measured temperature decrease in our device is fitted by an exponential decay with time-

constant τ = 2.23 min, with the temperature at the chip center reaching 80% of its steady-state value in ~4 min and 90% in ~6 min. The slight difference between the measured decay and the simulated one is mainly attributed to imperfections of the thermal contact among the different parts of the assembly, which are not included in the FEM calculations. Much faster cooling is obviously observed at the metallic frame, where the temperature reaches 90% of its final value within 2 min (Figure SI3).

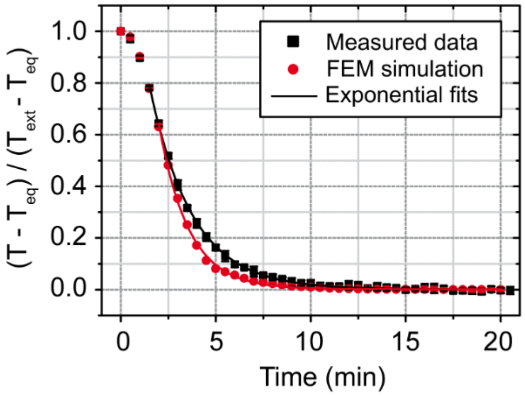

Figure SI2 Temporal evolution of the "normalized chip temperature". Experimental data are obtained by plotting the curves of Figure 3a normalized in the form of $(T - T_{eq})/(T_{ext} - T_{eq})$; each experimental point represents the average of all normalized data points for a specific time.

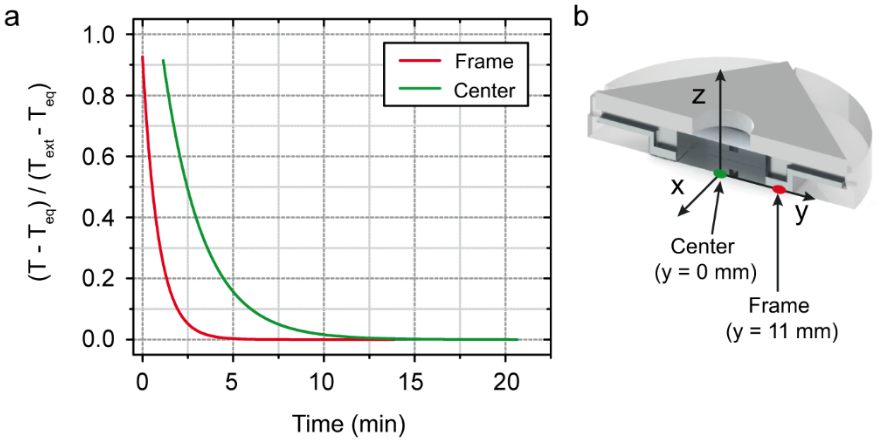

Figure SI3 (a) Exponential fits of experimental data of the normalized temperature $(T - T_{eq})/(T_{ext} - T_{eq})$ as a function of the cooling time. Measurements are taken by means of a Pt1000 sensor, in contact with the glass coverslip, positioned at the center of the chip ("Center") and below the metallic frame ("Frame") , as indicated in (b). The Frame position shows a much faster thermalization than the Center position, mainly related to the good thermal conductivity of the aluminum part, which is playing a crucial role in our thermal setup.

Supplementary Note 4. Inflow pre-thermalization study.

As previously described, the fluid entering the microfluidic chip is pre-thermalized during its passage through the metallic frame in which both PDMS chip and tubing are inserted. First, this allows achieving lower on-chip temperatures than without fluid flow, thereby improving the cooling efficiency of the device. Second, fluid pre-thermalization reduces the time constants of the system, thus making temperature changes faster and reducing the overall stabilization time of each experiment. Finally, the speed of the pre-thermalized inflow can be exploited as an additional parameter for temperature control, allowing the fine-tuning of the temperature of the actual microfluidic environment. We experimentally characterize the influence of the pre-thermalized inflow by measuring the steady-state temperature at the chip center for constant electrical power and different flow rates of the liquid entering the chip (Figure SI4a). For this study, we work at constant power $P \sim 24$ W, since this results in setting the on-chip temperature around 15 °C, which is a critical value for our applications, as will be shown later. In this case, fast inflows (> 1000 nL/s) allow a 25% further reduction of the chip temperature, down to about 11 °C. In certain experiments, such additional cooling by pre-thermalization may, however, be undesirable and can be rendered minimal by working at relatively low flow rates (e.g. 100-200 nL/s) or finely-tuned via proper combinations of thermoelectric and liquid cooling parameters. Finally, we characterize the dynamics of heat exchange during liquid injection at different flow rates: liquid pre-thermalization allows much faster chip cooling, e.g. resulting in a 44% to 87% reduction of the time needed to reach 15 °C at $P \sim 24$ W, for flow rates from 250 to 2000 nL/s, respectively (Figure SI4b).

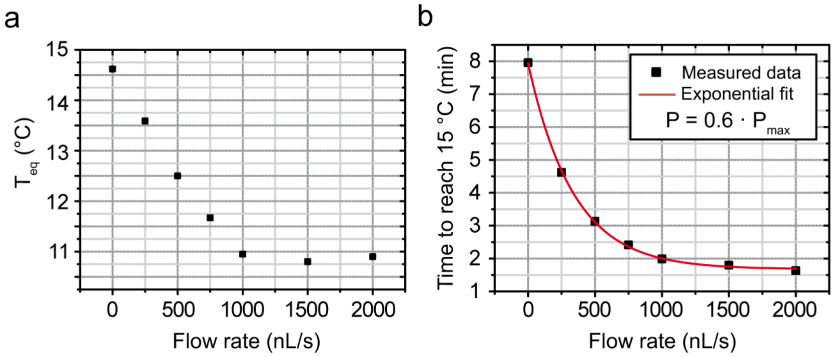

Figure SI4 (a) Measured equilibrium temperature T_{eq}, and (b) time to reach T = 15 °C during liquid injection at different flow rates at constant cooling power ($P = 0.6 \times P_{max} \sim 24$ W, sensor at chip center).

Supplementary Note 5. Worm viability and culture tests.

Before using our device for specific analytical studies, we test its capability of automatically running worm cultures over several days and optimize the worm feeding protocol for good worm viability and healthy growth. For this preliminary validation, we employ wild-type *C. elegans* and feed them with HT115 *E. coli* bacteria. Different feeding protocols are tested to establish optimal conditions for worm growth. A delicate balance has to be found between two main competing factors. On the one hand, too strongly diluted *E. coli* suspensions (and/or less frequent injections) might cause worm starvation. On the other hand, too concentrated *E. coli* suspensions (and/or too frequent injections) usually result in the uncontrolled clogging of narrow microfluidic connections, which has then to be eliminated via user-controlled washing steps. A good compromise between these factors is found by injecting in the device ~1.5 µL *E. coli* suspension per microfluidic chamber (e.g. ~6 µL for a 4-chamber matrix) at 1.4×10^9 bacteria/mL concentration, every 10 minutes. This protocol is automatically handled by the syringe pump software interface and allows attaining reliable worm growth inside the chip over several days, with no significant channel clogging or extra washing steps (Figure SI5). Moreover, worm immobilization via thermoreversible PF127 gelation proves neither to affect worms' viability nor to alter their physiological functions, as already reported in previous works.[1-4]

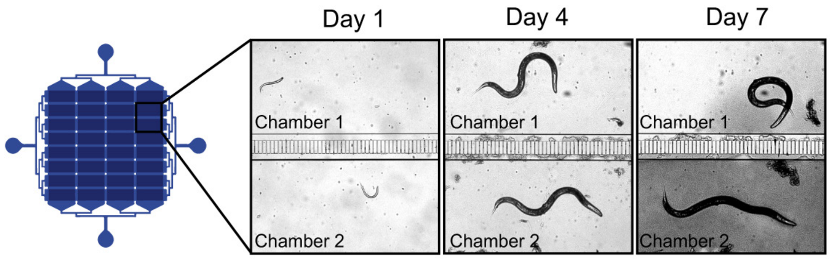

Figure SI5 Experimental validation of worm culture inside two representative microfluidic chambers of the matrix. Experiments are performed using the chip design reported in Fig.1diii of the main text, which is tailored for the loading of worms at the L1 stage, in order to be able to follow the worms' development starting from their first larval stage. We report here representative time-lapse pictures of two culture chambers, containing isolated wild-type worms. We demonstrate reliable on-chip worm maintenance and reproducible worm growth over 7 days, moreover confirming the possibility to keep single worms confined in separate chambers over their whole lifespan.

Supplementary Note 6. Protein aggregation analysis in a Huntington disease *C. elegans* model.

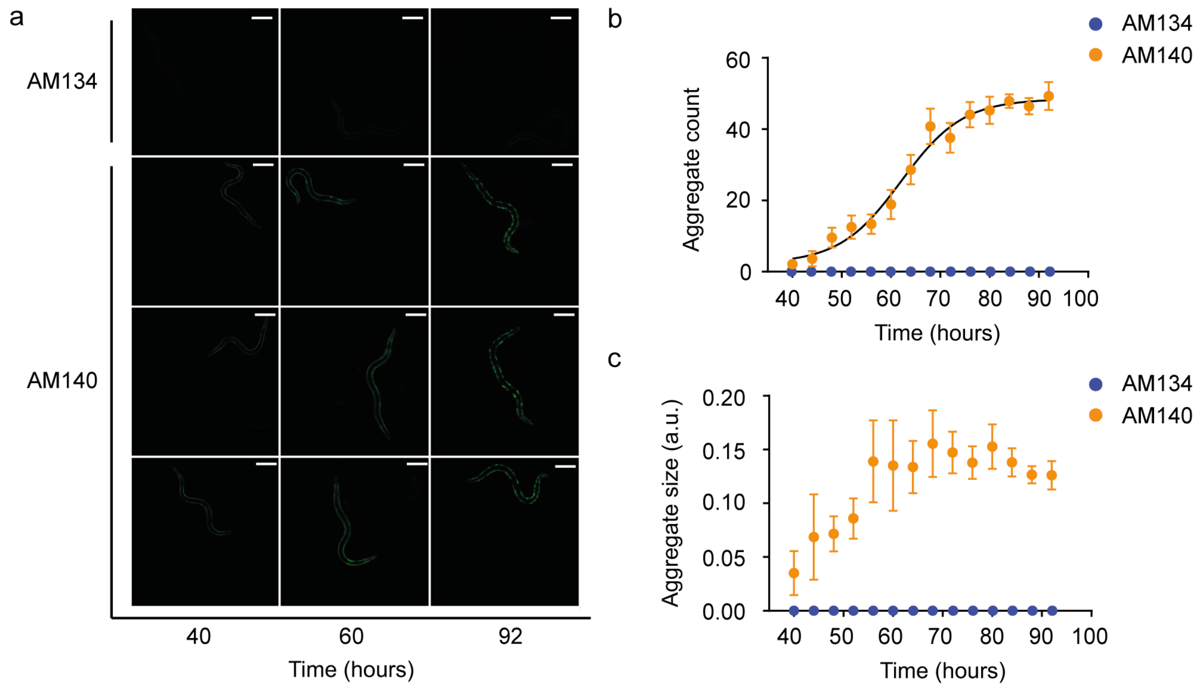

Figure SI6 (a) Time-lapse fluorescent pictures of three representative AM140 transgenic worms, used for the study of polyQ aggregation in muscle cells, and one representative AM134 worm, used as a negative control for the analysis. Scale bars = 200 µm. (b-c) Temporal evolution of (b) the average number and (c) the average size of the aggregates counted per each worm, as obtained from the image processing algorithm over the period from 40 to 92 hours upon worm loading on chip (day 1 to day 3 of worm adulthood). Graphs are expressed as mean + SEM (N=9).

References:

(1) Krajniak, J.; Lu, H. *Lab on a chip* **2010**, *10*, 1862-1868.

(2) Krajniak, J.; Hao, Y.; Mak, H. Y.; Lu, H. *Lab on a chip* **2013**, *13*, 2963-2971.

(3) Hwang, H.; Krajniak, J.; Matsunaga, Y.; Benian, G. M.; Lu, H. *Lab on a chip* **2014**, *14*, 3498-3501.

(4) Aubry, G.; Zhan, M.; Lu, H. *Lab on a chip* **2015**, *15*, 1424-1431.
